# Pharmacogenetic CYP2B6 variants affect steroid hormone metabolism in human breast cancer cells

**DOI:** 10.1002/bcp.70473

**Published:** 2026-02-04

**Authors:** Marco Hoffmann, Julian Peter Müller, Stefan Düsterhöft, Sabrina Yamoune, Katja Susanne Just, Julia Carolin Stingl

**Affiliations:** ^1^ Institute of Clinical Pharmacology University Hospital RWTH Aachen Aachen Germany; ^2^ Institute of Molecular Pharmacology University Hospital RWTH Aachen Aachen Germany; ^3^ Department of Clinical Pharmacology and Pharmacoepidemiology University Hospital Heidelberg Heidelberg Germany

**Keywords:** breast cancer, CYP2B6, drug metabolizing enzymes, gender‐sensitive pharmacology, pharmacogenetics, steroid metabolism

## Abstract

**Aims:**

CYP2B6 is a key enzyme involved in the metabolism of steroid hormones such as testosterone and estradiol. Common genetic CYP2B6 variants (*4, *5, *6, *9) are associated with reduced enzymatic activity and have been linked to increased breast cancer risk and poor prognosis. However, the impact of these genetic variants on testosterone and estradiol metabolism in humans is not understood. Therefore, this study aimed to investigate how these pharmacogenetic CYP2B6 variants affect metabolism of these steroid hormones in a human breast cancer model and how this may contribute to altered steroid hormone profiles in breast cancer.

**Methods:**

T47D breast cancer cells were engineered to stably overexpress CYP2B6 wild type and the variants *4, *5, *6 and *9 using a retroviral pMOWS vector system. The metabolites 16α‐/16β‐hydroxytestosterone and 2‐/4‐hydroxyestradiol were analysed using HPLC‐MS/MS after incubation of testosterone or estradiol with CYP2B6 and CYP1B1 supersomes and the modified T47D cells. Conversion of testosterone metabolites to oestrogens by aromatase was also tested.

**Results:**

CYP2B6 supersomes predominantly formed 16β‐hydroxytestosterone and 2‐hydroxyestradiol, while CYP1B1 predominantly produced 16α‐hydroxytestosterone and 4‐hydroxyestradiol. CYP2B6*6 overexpression in T47D increased 16α‐hydroxytestosterone formation, while *4 and 9 showed decreased metabolism compared to wild type. CYP2B6*5 produced reduced 16β‐hydroxytestosterone levels. Aromatase converts 16α‐metabolite to estriol and 16β‐hydroxytestosterone to 16‐epiestriol.

**Conclusions:**

This study demonstrates that common CYP2B6 variants alter testosterone metabolism in a human breast cancer model, potentially disrupting steroid hormone balance and contributing to a tumour‐promoting environment. These findings highlight the potential relevance of pharmacogenetic profiling in breast cancer risk assessment.

## Graphical abstract

Common genetic CYP2B6 variants correlate with adverse breast cancer outcome. The oestrogen metabolites estriol and 16‐epiestriol, which are formed downstream of CYP2B6‐catalysed 16α/β‐hydroxytestosterone, may be linked to elevated breast cancer risk and might increase due to the demonstrated CYP2B6 variants‐related metabolic shifts.

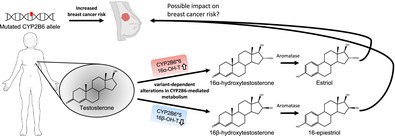

**What is already known about this subject?**

- CYP2B6 metabolizes testosterone and estradiol, and common variants are associated with increased breast cancer risk.
-
*E. coli* studies indicate that frequent CYP2B6 variants alter testosterone metabolism outside an intact cellular environment.
- No study addressed human CYP2B6*5 and the effects of these common variants in a human model remain unknown.

**What this study adds?**

- In breast cancer cells, human CYP2B6*5 reduces 16β‐hydroxylation, whereas CYP2B6*6 increases 16α‐hydroxylation of testosterone.
- These common human CYP2B6 variants may alter steroid hormone balance and contribute to breast cancer risk.
- Therefore, pharmacogenetic profiling of *CYP2B6* could be relevant for individual risk assessment.

## 1 INTRODUCTION

Cytochrome P450 2B6 (CYP2B6) is a human drug‐metabolizing enzyme involved in the metabolism of about 8% of clinically applied drugs.^1^ In addition to its predominant hepatic expression, CYP2B6 is found in extrahepatic, steroid hormone‐sensitive tissues such as the prostate and breast.^2^, ^3^ Current evidence suggests that in general, CYP2B6 may have a protective role in breast cancer (BC) development: Local CYP2B6 expression correlates with prolonged disease‐free survival in BC patients.^4^ However, the allelic variants CYP2B6*4 (rs2279343) and CYP2B6*9 (rs3745274), as well as their combined haplotype CYP2B6*6, correlate with higher risk for steroid hormone‐dependent prostate cancer^5^ and BC.^6^ Additionally, CYP2B6*5 (rs3211371) has been linked to poor prognosis in hormone receptor‐positive BC, especially in premenopausal patients.^7^ Besides the wild type, CYP2B6*9, *6, *5 and *4 are, in descending order, the most common alleles worldwide.^8^, ^9^ Although these variants generally are known to exhibit reduced catalytic activity, their impacts are highly substrate‐dependent^10^ and, in some cases, may even result in increased activity.^11^, ^12^, ^13^

Beyond drug metabolism, CYP2B6 has also been identified as an enzyme involved in the metabolism of steroid hormones, particularly testosterone (T)^14^ and estradiol (E2),^15^ both of which have been linked to increased BC risk.^16^, ^17^, ^18^ CYP2B6 mediates both 16α‐ and 16β‐hydroxylation of T.^14^ Studies using *Escherichia coli* (*E. coli*)‐expressed variants demonstrated that CYP2B6*4, *6 and *9 show reduced 16α‐hydroxylation activity, whereas CYP2B6*6 exhibits increased 16β‐hydroxylation compared to wild type.^11^ Similarly, the human‐like cynomolgus‐derived CYP2B6*5 variant showed reduced T 16β‐hydroxylation.^19^ Importantly, the 16α‐hydroxylated metabolite, unlike 16β‐hydroxy‐T (16β‐OH‐T), is known to be a substrate of aromatase, which converts it to estriol (E3).^20^ To date, only one study has examined the role of CYP2B6 in E2 metabolism, reporting very low catalytic activity, with weak 2‐hydroxylation of E2 and estrone and negligible 4‐hydroxylation.^15^

In contrast, CYP1B1 is a major enzyme in the extrahepatic metabolism of estradiol (E2) and other steroid hormones.^21^ It primarily forms the carcinogenic 4‐hydroxylated E2 metabolite (4‐OH‐E2),^22^ and also 16α‐OH‐T from T, but not 16β‐OH‐T.^21^ Elevated CYP1B1 expression^23^, ^24^ and increased 4‐OH‐E2 levels^25^, ^26^ have both been associated with higher BC risk and poor prognosis. 4‐OH‐E2 is considered more carcinogenic than 2‐OH‐E2 due to its instability and formation of reactive quinones that can cause DNA damage and mutations.^27^ Thus, CYP2B6 and CYP1B1 may differentially influence hormone‐sensitive BC development through their distinct metabolite profiles, with CYP2B6 potentially exerting a protective, opposing effect. To date, E2 metabolism by CYP2B6 variants has not been studied at all, and studies on T metabolism have been limited to expression in *E. coli* systems^11^, ^19^ or to non‐human variants.^19^

Therefore, this study aimed to investigate the influence of the most common and BC‐associated CYP2B6 variants on steroid hormone metabolism by analysing their impact on T and E2 biotransformation in intact human BC cells. For the first time, by overexpression of CYP2B6*1 (wild type), *4, *5, *6 and *9 in the T47D BC cell line, we were able to describe variant‐specific differences in T metabolism in a human hormone‐sensitive model. We characterized metabolite profiles and compared metabolite formation rates between the variants. By elucidating these metabolic pathways, our study provides further insight into the role of CYP2B6 in hormone‐dependent BC.

## 2 MATERIAL AND METHODS

### 2.1 Materials

T47D cells (HTB‐133) were purchased from ATCC (Manassas, Virginia, USA) and authenticated by STRS analysis. Testosterone (86500), estradiol (E2758), L‐ascorbic acid (A4544), 4‐OH‐E2 (H4637), estriol (E1253) and phenylmethylsulfonylfluorid (PMSF) (10837091001) were from Sigma Aldrich (St. Louis, Missouri). 2‐OH‐E2 (13019‐1) was purchased from Cayman Chemicals (Ann Arbor, Michigan, USA). 16‐Epiestriol (Cay33455‐10) was from Biomol (Hamburg, Germany). Metabolites 16α‐ (AAA06301) and 16β‐OH‐T (3D‐SAA52890) were purchased from Biosynth (Staad, Switzerland). Information on all used materials can be found in Supporting Information S1.

### 2.2 Testosterone and estradiol metabolism by isolated CYP2B6 and CYP1B1

To examine the specific metabolite profiles of T and E2, 140 pmol (for T) or 70 pmol (for E2) of CYP2B6 (Order no.: 456210) or CYP1B1 (Order no.: 456220) supersomes (Discovery Life Sciences, Kassel, Germany) were incubated for 1 h with 100 μM of T or 20 μM of E2. Samples were measured by HPLC‐MS/MS. A detailed method description is available in supplementary file 1.

### 2.3 pMOWS vector cloning for T47D cell transduction

To generate a stable overexpression of different CYP2B6 variants in T47D cells, the pMOWS vector system^28^ was utilized, expressing the green fluorescence protein (GFP) as reporter gene. cDNA sequences of CYP2B6*1, *4, *6 and *9 were cloned from a pcDNA3.1 vector,^29^ and CYP2B6*5 cDNA was newly generated using a mutagenic primer. After purification the vectors and inserts were assembled and the constructs were transformed into *E. coli* (Order no.: C3040I, NEB), single colonies were picked, and DNA was isolated. Constructs were sequenced by Eurofins Genomics using Sanger sequencing. A detailed method description is available in Supporting Information S1.

### 2.4 Generation of stable T47D cells overexpressing GFP or CYP2B6 variants

Using the Phoenix‐AMPHO retrovirus producer cell line, the retroviruses containing the respective target gene sequences (*GFP*, CYP2B6*1, *4, *5, *6 or *9) were produced. Therefore, the cells were seeded and transfected using Lipofectamine 3000 (Order no.: L3000015, ThermoFisher Scientific) with the generated pMOWS vector constructs. The retrovirus containing medium was collected and filtered, before it was added to freshly seeded T47D cells for transduction. Selection of isogenic cells was performed using 0.5 μg/mL of puromycin. Medium was exchanged every 2–3 days, and all cells were cultured at 37°C and in a 5% CO_2_ environment. For a more detailed method description see Supporting Information S1.

### 2.5 Quantitative real‐time polymerase chain reaction (qPCR) measurement of *CYP2B6* overexpression in T47D cells

qPCR was performed to verify the overexpression of the *CYP2B6* variants on mRNA level in the transduced T47D cells. Therefore, the cells were seeded and harvested with *TRIzol™* reagent (Order no.: 15596‐018, ThermoFisher Scientific) after 24 h to perform RNA isolation. For cDNA synthesis, the *High‐Capacity cDNA Reverse Transcription Kit* (Order no.: 4368814, ThermoFisher Scientific) was utilized and qPCR was performed using *PowerUP SYBR Green* (Order no.: A25776, ThermoFisher Scientific) with *QuantiTect Primer Assays* (QIAGEN, Venlo, Netherlands) for the genes of phosphoglycerate kinase 1 (*PGK1)* (Order no.: QT00013776) and *CYP2B6* (Order no.: QT00000910). For normalization of the *CYP2B6* expression, the *PGK1* expression was used.

### 2.6 Western blot analysis of CYP2B6 overexpression in T47D cells

To validate CYP2B6 expression in the transduced T47D cells on the protein level, western blot analysis was performed. For this, the cells were seeded and lysed after 24 h. After denaturation, the protein samples, and CYP2B6 supersomes as positive control, were separated by SDS‐PAGE and transferred onto a PVDF membrane. For chemiluminescent detection of CYP2B6, the primary rabbit anti‐human polyclonal CYP2B6 antibody (Order no.: 70R‐32 408, Biosynth, Staad, Switzerland) and secondary goat anti‐rabbit IgG horseradish‐peroxidase (HRP) antibody (Order no.: 31460, ThermoFisher Scientific) were used. For normalization, the measurement of glycerinaldehyde 3‐phosphat dehydrogenase (GAPDH) protein expression took place utilizing the primary mouse anti‐human monoclonal antibody (Order no.: 649202, Biolegend, San Diego, CA) and the secondary goat anti‐mouse IgG HRP antibody (Order no.: 31569, ThermoFisher Scientific). Western blot was repeated three times with different cell passages. Using the ‘Sum of the Replicate’ method described by Degasperi et al.,^30^ the mean relative expression ratio of the CYP2B6 variants was calculated. This ratio was used to normalize the T metabolism experiments in T47D cells. For a more detailed method description, see Supporting Information S1.

### 2.7 Testosterone metabolism in T47D cells overexpressing CYP2B6 variants

The same cell passages used for western blot experiments were used for T metabolism measurements. Cells were seeded and after 24 h 100 μM of T was added to start the reaction. The supernatant was collected after 1, 4, 8 and 16 h and samples were measured by HPLC‐MS/MS. For metabolite level normalization on total protein amount, a bicinchoninic acid (BCA) assay was performed. To compare the activity of the CYP2B6 variants, the relative metabolite formation rate was calculated by division of the T metabolism rate (pmol/min) by the CYP2B6 protein expression ratio measured via western blot. A more detailed method description is available in Supporting Information S1.

### 2.8 Bicinchoninic acid (BCA) assay of T47D samples

Normalization on total protein amount of the measured T metabolite values from the cell experiments was performed by BCA assay (Order no.: 23225, ThermoFisher Scientific). A detailed method description is available in Supporting Information S1.

### 2.9 Conversion of testosterone metabolites to estriol or 16‐epiestriol by aromatase

To examine the metabolism of 16α‐ and 16β‐OH‐T to E3 or 16‐epiestriol by aromatase, 70 pmol of aromatase supersomes (Order no.: 456260, Discovery Life Sciences) were incubated with 20 μM of 16α‐ and 16β‐OH‐T for 1 h and measured via HPLC‐MS/MS. For a more detailed method description, see Supporting Information S1.

### 2.10 High performance liquid chromatography–tandem mass spectrometry (HPLC‐MS/MS) measurements of steroid hormone metabolites

HPLC‐MS/MS was utilized for quantification of the metabolite levels from all metabolism experiments. The analyte peaks were quantified using external calibration curves with at least 5 non‐zero standards in sample matrix. A detailed description of the different HPLC‐MS/MS measurements and methods is presented in Supporting Information S1.

### 2.11 Statistical analysis

For the comparison of T 16‐hydroxylation values across different CYP2B6 variants in T47D cells, the metabolite formation of each variant was compared to that of the wild type (CYP2B6*1). Statistical analyses were performed using one‐way ANOVA with matched pairs, corrected for multiple comparisons using the Holm‐Šidák test. Normality was verified using the Shapiro–Wilk test. Variability assumptions, including sphericity and homoscedasticity, were tested. The data are expressed as mean ± SD and were derived from three independent experiments. Significance levels are denoted by asterisks with *p* ≤ 0.05 indicated by *, *p* ≤ 0.005 by ** and *p* ≤ 0.001 by ***, representing increasing levels of statistical significance. Statistical analyses and graphical representations were performed using GraphPad Prism® 10.

### 2.12 Nomenclature of targets and ligands

Key protein targets and ligands in this article are hyperlinked to corresponding entries in http://www.guidetopharmacology.org and are permanently archived in the Concise Guide to PHARMACOLOGY 2023/24.^31^, ^32^, ^33^

## 3 RESULTS

### 3.1 Testosterone and estradiol hydroxylation by isolated CYP2B6 compared to CYP1B1

Since high CYP1B1^23^, ^24^ but low CYP2B6 levels^4^ are related to BC, as a first step, we wanted to directly compare the specific metabolite profile of T and E2 generated by isolated CYP2B6 and CYP1B1.

**FIGURE 1:**
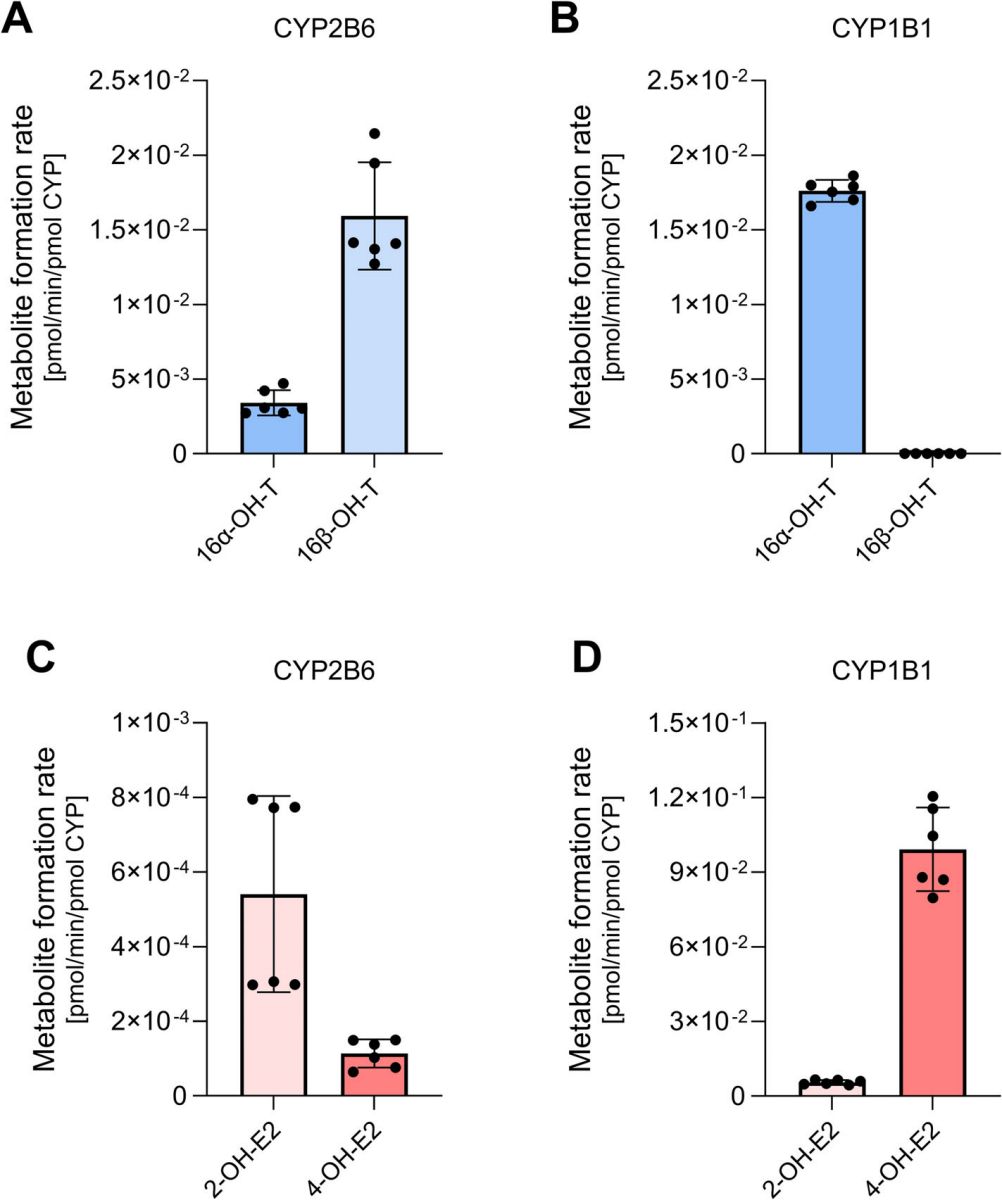
Determination of metabolite formation rates with metabolite profile of testosterone and estradiol by recombinant CYP2B6 and CYP1B1. CYP2B6 or CYP1B1 supersomes were incubated with 100 μM testosterone (T) (140 pmol supersomes) (A, B) or 20 μM estradiol (E2) (70 pmol supersomes) (C, D), 2 mM NADPH and in case of E2 with 5 mM ascorbic acid for metabolite stabilization for 1 h at 37°C. Reaction was stopped by diluting 1/5 with methanol, and the supernatant was analysed by HPLC‐MS/MS. Mean of metabolite formation rate ± SD from two independent experiments with three biological replicates each are shown.

To measure the respectively formed metabolites 16α/β‐OH‐T of T and 2/4‐OH‐E2 of E2 by isolated CYP2B6 and CYP1B1 supersomes, we incubated each enzyme for 1 h with T or E2 and quantified the metabolite levels via HPLC‐MS/MS. We found that isolated CYP2B6 enzyme formed both 16α‐OH‐T and 16β‐OH‐T (Figure 1A), while for 16β‐OH‐T, the formation rate was 4.6 times higher. Incubation of isolated CYP1B1 with T led to formation of 16α‐OH‐T but not 16β‐OH‐T (Figure 1B). E2 metabolism by CYP2B6 led to formation of both 2‐ and 4‐OH‐E2, with a 4.8‐fold higher metabolite formation rate for 2‐OH‐E2 over 4‐OH‐E2 (Figure 1C). CYP1B1 showed a much higher E2 metabolism with a 10.2‐fold formation of 2‐OH‐E2 and 875.8‐fold production of 4‐OH‐E2 from E2, compared to CYP2B6 (Figure 1C,D). The ratio between 4‐OH‐E2 and 2‐OH‐E2 formation by CYP1B1 was 19‐fold.

### 3.2 Validation of CYP2B6 variants overexpression in T47D cells

**FIGURE 2:**
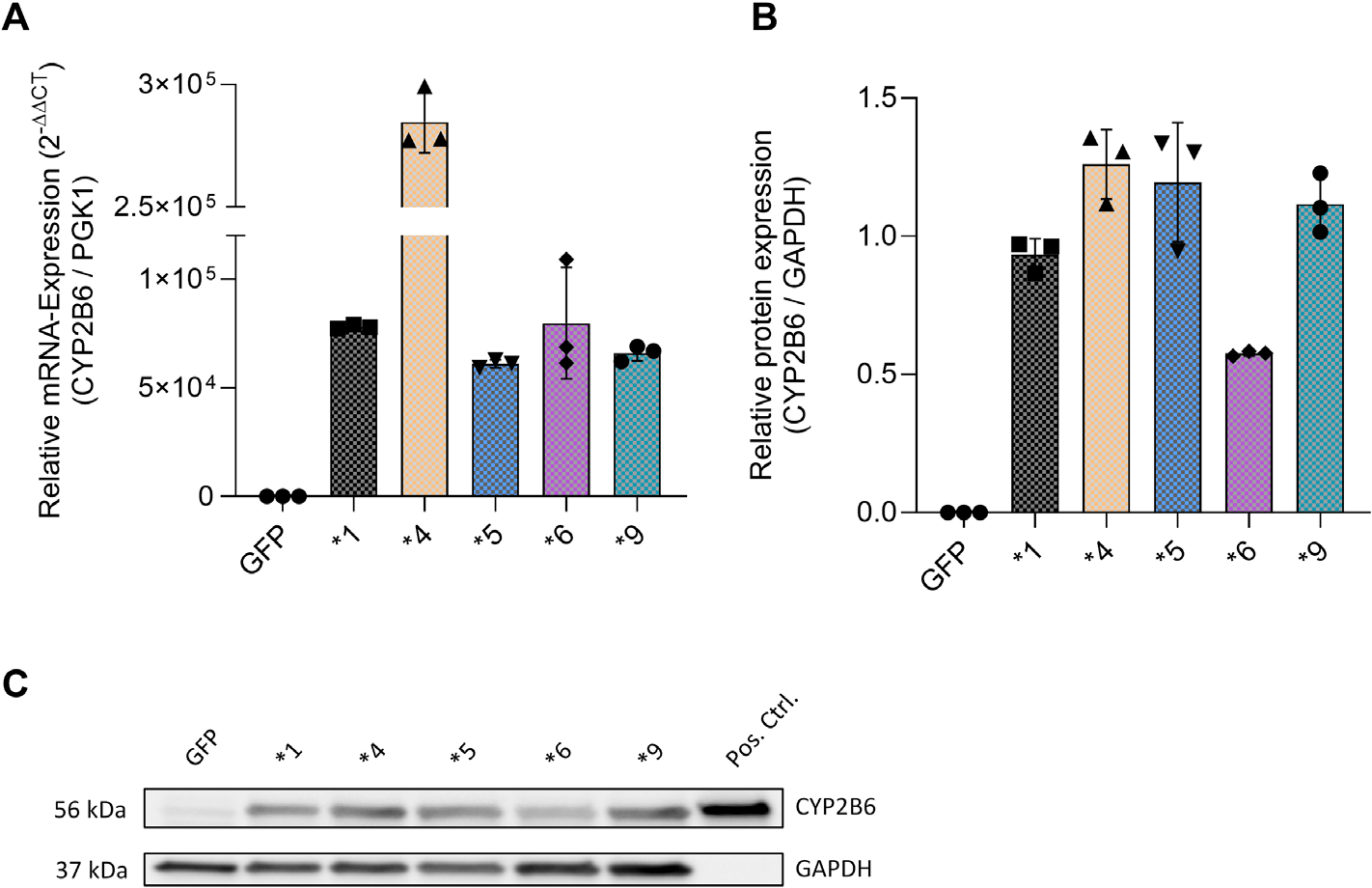
Validation of the T47D overexpression system for CYP2B6*1, *4, *5, *6 or *9 by qPCR analysis and western blot. T47D cells overexpressing GFP or a CYP2B6 variant were lysed and CYP2B6 expression was measured on mRNA level by qPCR (A) and on protein level by western blot (B, C). Relative mRNA expression is shown as mean values of three technical replicates ± SD normalized to reference gene PGK1 and compared to GFP control. Relative western blot data from three independent experiments are shown as mean ± SD. Protein expression values were normalized to GAPDH as reference protein. CYP2B6 variants expression ratio was determined by dividing each band intensity by the sum of all bands for each western blot. A representative western blot of T47D cells overexpressing GFP or CYP2B6 variants and 0.5 pmol of CYP2B6 supersomes as positive control (Pos. Ctrl.) is shown (C).

To get a deeper understanding of the connection between CYP2B6 variants and BC, we aimed to investigate the variants specific changes in the metabolism of T and E2 in a human model. To create a clinically relevant hormone‐sensitive BC model, we established human T47D cells overexpressing the variants CYP2B6*4, *5, *6 and *9, the wild type enzyme and GFP as a reporter. Thereby we include factors potentially influencing enzyme activity, such as post‐translational modifications as well as transport and regulatory processes. For production of the cell lines, the retroviral pMOWS vector system was used,^34^ and the overexpression was validated by qPCR and western blot. The latter was also used for expression ratio determination of the CYP2B6 variants and for normalization of metabolism measurements in T47D cells. The qPCR data of T47D cells overexpressing GFP, CYP2B6*1, *4, *5, *6 or *9 are shown in Figure 2A. Cells with *GFP* expression showed low basal levels of *CYP2B6* mRNA expression, while cells transduced with *CYP2B6* variants expressed at least 60 996‐fold *CYP2B6* mRNA compared to the *GFP* control. Western blot analysis of these cell lines demonstrated successful overexpression of all CYP2B6 variants on protein level with a band signal at 56 kDa (Figure 2B). Since in the GFP control the signal was not quantifiable, no relative value of the CYP2B6 protein expression compared to the GFP control could be determined. Figure 2C shows a representative western blot.

### 3.3 Testosterone metabolism in T47D cells overexpressing CYP2B6 variants

**FIGURE 3:**
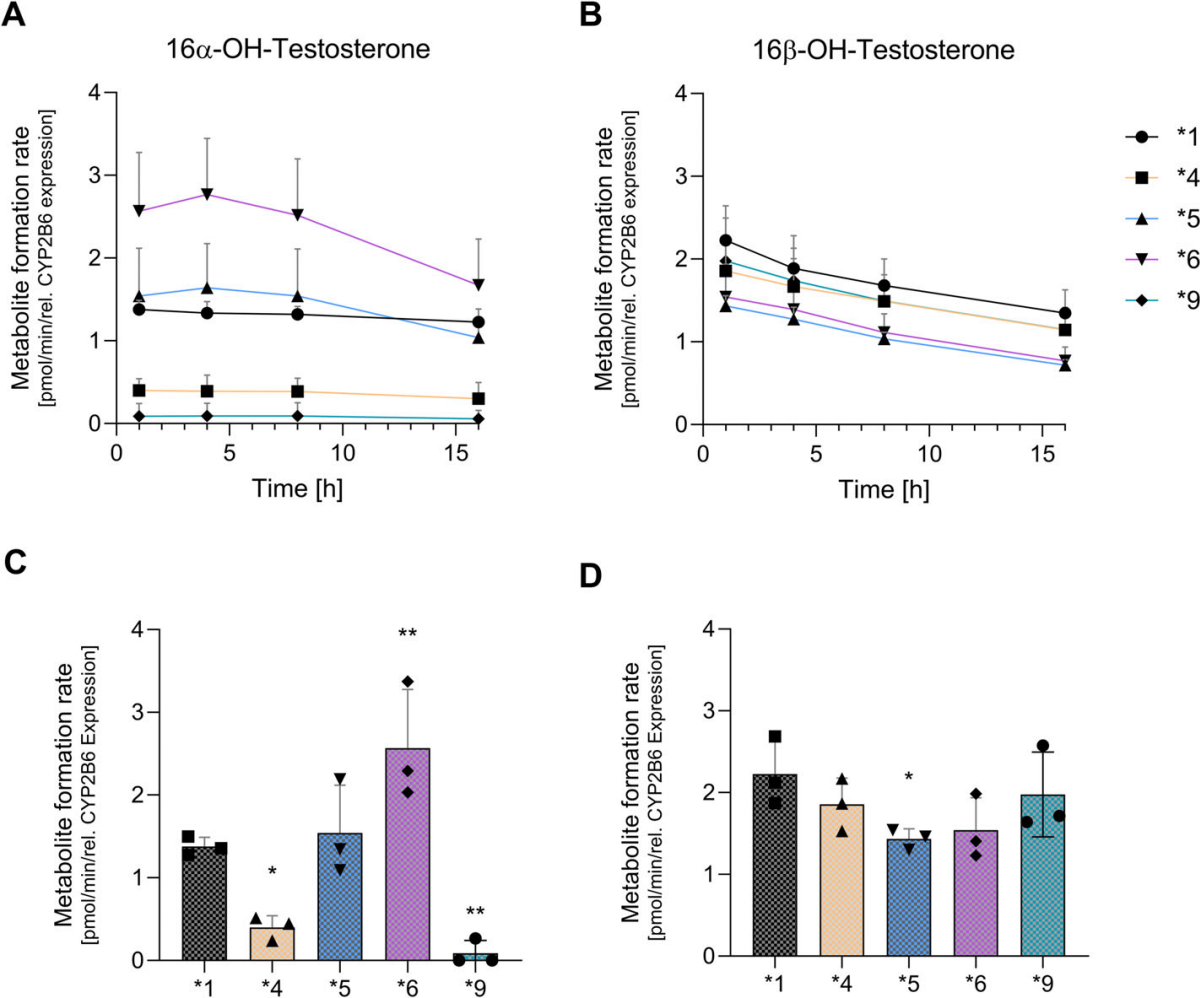
Metabolism of testosterone by T47D cells overexpressing CYP2B6 variants. GFP, CYP2B6*1, *4, *5, *6 or *9 overexpressing T47D cells were incubated with 100 μM testosterone (T). Supernatant was collected after 1, 4, 8 and 16 h, and 16α‐ (A) and 16β‐hydroxytestosterone (16α/β‐OH‐T) (B) were measured via HPLC‐MS/MS. After 1 h of T incubation, 16α‐ (C) or 16β‐OH‐T (D) are shown separately. Mean of metabolite formation rate normalized to total protein content and CYP2B6 expression ratio from western blot analysis ± SD from three independent experiments is shown. Before normalization on CYP2B6 expression, background levels of 16α‐OH‐T formation (GFP control) were subtracted from values of CYP2B6 variant overexpressing cells. Each column was compared to column of variant *1 and statistically analysed using one‐way ANOVA with GraphPad Prism 10. *p* < 0.05: *, *p* < 0.01: **.

To investigate the metabolite formation rates of T to 16α‐ and 16β‐OH‐T by the human pharmacogenetic CYP2B6 variants *4, *5, *6 and *9 compared to wildtype (CYP2B6*1), the established human T47D BC cells with stable overexpression were used. We also aimed to measure CYP2B6 variant specific differences in 2‐ and 4‐hydroxylation of E2. Compared to GFP control cells, we were not able to detect higher levels of these metabolites in the CYP2B6‐overexpressing cells and therefore could not quantify the CYP2B6‐mediated E2 metabolism. The cells were incubated with 100 μM T for 1, 4, 8 and 16 h, and metabolite formation was measured by HPLC‐MS/MS (Figure 3A,B). Since at the time‐point of 1 h the metabolite formation rate was the highest for most reactions, we decided to take a closer look at the specific activity differences of the variants at this time point (Figure 3C,D). Concerning 16α‐OH‐T (Figure 3C), T47D cells overexpressing GFP showed minor formation of this metabolite, which was regarded as background signal and subtracted from the concentrations measured with each CYP2B6 variant. For evaluation of the variant specific differences in T metabolite formation, the obtained metabolite levels were normalized to the total protein amount determined by BCA‐assay. To calculate the metabolite formation rates of the variants, the metabolite generation per min was divided by the calculated CYP2B6 expression ratio from the western blot experiments. We found that T47D cells overexpressing CYP2B6*4 and *9 showed a reduced, while CYP2B6*6 expression revealed an increased metabolite formation rate of T for the 16α‐hydroxylation reaction compared to the wild type CYP2B6*1 (Figure 3C). Regarding 16β‐OH‐T formation we found a significantly reduced metabolite formation rate by T47D cells overexpressing CYP2B6*5 compared to the wild type (Figure 3D).

### 3.4 Conversion of testosterone metabolites to oestrogens by aromatase

**FIGURE 4:**
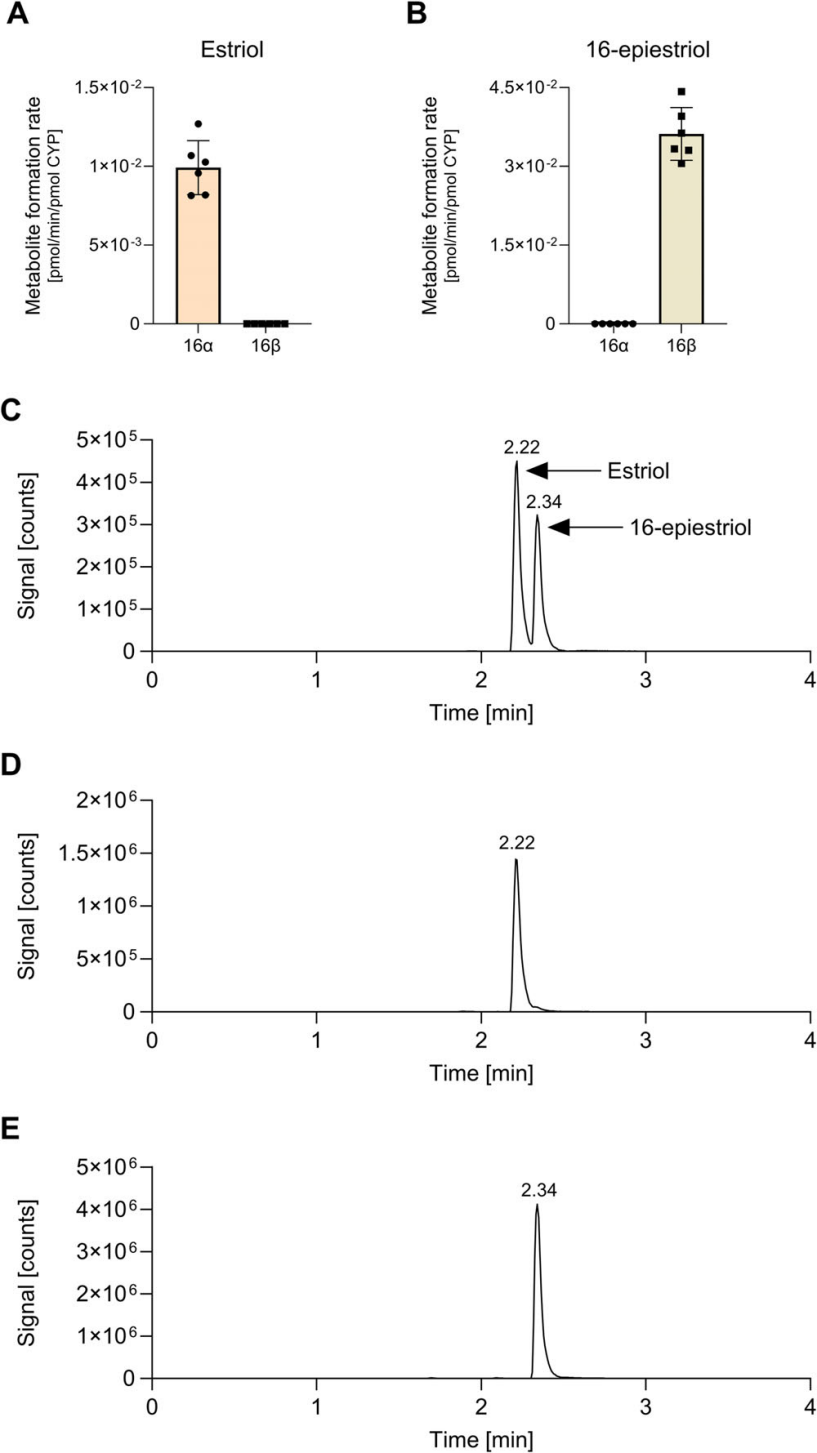
Metabolite formation rates of testosterone metabolites to oestrogens by recombinant aromatase. 20 μM of 16α‐ or 16β‐OH‐T was incubated with 70 pmol of isolated aromatase for 1 h, and estriol (A) and 16‐epiestriol (B) formation was measured via HPLC‐MS/MS. Mean of metabolite formation rate ± SD from two independent experiments with three biological replicates each is shown. The chromatograms show the specific peaks for estriol and 16‐epiestriol (mass transition 287 ➔ 171 *m*/*z*) of 50 ng/mL of both standards (C), of a sample incubated with 16α‐OH‐T (D) and of a sample incubated with 16β‐OH‐T (E).

CYP2B6*6 and *5 are associated with increased BC risk^6^ or poor prognosis,^7^ and we observed changes in 16‐hydroxylation profiles of T by cells overexpressing these variants towards higher 16α‐ and lower 16β‐OH‐T formation (Figure 3C,D). Therefore, we aimed to investigate whether these metabolism changes might lead to altered oestrogenic activity by aromatization. To elucidate the potential of these two metabolites to be converted to oestrogens by aromatase, we used isolated aromatase incubated with 16α‐ or 16β‐OH‐T for 1 h. We confirm that 16α‐OH‐T is converted to E3, while 16β‐OH‐T was metabolized to the stereoisomer of E3 termed 16‐epiestriol, which has not been described before (Figure 4).

## 4 DISCUSSION

This is the first study to quantify T biotransformation mediated by human CYP2B6*1, *4, *5, *6 and *9 in a cellular context using intact human T47D BC cells stably overexpressing these variants. Overexpression of CYP2B6*6 increased, while CYP2B6*4 and *9 decreased the metabolite formation rate of T to 16α‐OH‐T compared to wild type. In addition, CYP2B6*5 reduced 16β‐OH‐T formation. These findings indicate that common CYP2B6 variants can alter T metabolism and potentially shift steroid hormone homeostasis towards a BC‐promoting state.

To emphasize the CYP2B6‐specific metabolite formation profiles of T and E2 in comparison to CYP1B1, a key steroid hormone‐metabolizing enzyme associated with BC risk,^23^, ^24^ we analysed 16‐hydroxylation of T and 2‐ and 4‐hydroxylation of E2 using supersomes. CYP2B6 predominantly formed 16β‐OH‐T, while CYP1B1 produced 16α‐OH‐T but not 16β‐OH‐T. This aligns with prior reports showing 6β‐, 15α‐ and 16α‐hydroxylation by CYP1B1^21^ and dual 16α/β‐hydroxylation by CYP2B6.^11^ For E2, CYP2B6 favoured 2‐OH‐E2 formation over 4‐OH‐E2, whereas CYP1B1 strongly preferred 4‐hydroxylation, exhibiting a 10.2‐fold higher 2‐OH‐E2 and an 875.8‐fold higher 4‐OH‐E2 production than CYP2B6. This underlines the dominant role of CYP1B1 in E2 metabolism, consistent with Lee et al., who observed weak 2‐OH‐E2 but no 4‐OH‐E2 formation by CYP2B6.^15^ Given the link between 4‐OH‐E2 and increased BC risk,^25^, ^26^ and the potential protective effect of 2‐OH‐E2,^35^ the opposing E2 metabolite profiles of CYP1B1 and CYP2B6 align with their contrasting associations with BC: CYP1B1 with increased risk,^23^, ^24^ and CYP2B6 with longer disease‐free survival and reduced proliferation marker levels in BC patients.^4^

In CYP2B6*6‐overexpressing T47D cells, 16α‐OH‐T formation was significantly increased, while 16β‐OH‐T formation tended to decrease compared to wild type. Similar metabolite shifts were reported by Lin et al. for another variant, showing increased 16α‐ and decreased 16β‐hydroxylation.^36^ We show that CYP2B6*4 and *9, both SNPs of *6, reduced 16α‐OH‐T formation, which aligns with studies in *E. coli*.^11^ However, our findings differ from Sridar et al., who observed reduced 16α‐OH‐T and increased 16β‐OH‐T formation for CYP2B6*6 after *E. coli* expression.^11^ This discrepancy likely stems from differences between expression systems. Intact human T47D cells offer a more physiologically relevant model than *E. coli*, accounting for post‐translational modifications, protein folding, cellular transport and regulatory processes, potentially affecting enzyme activity. They also reflect hormone receptor‐positive BC cell characteristics, enhancing clinical relevance. In the case of CYP2B6*5, we observed reduced 16β‐OH‐T formation, consistent with findings from Uno et al. for the cynomolgus‐derived CYP2B6*5 variant (91% sequence identity^37^) expressed in *E. coli*.^19^

In E2 metabolism investigations with CYP2B6‐overexpressing cells, no 2‐OH‐E2 or 4‐OH‐E2 levels above the GFP control were detected. This supports previous findings that CYP2B6 plays only a minor role in E2 metabolism compared to CYP1B1,^15^, ^21^ as illustrated in Figure 1. Hence, CYP2B6‐mediated T metabolism may be more relevant than E2 metabolism regarding its role in BC. However, it remains unclear whether CYP2B6‐derived T metabolites directly influence disease progression or explain the observed associations between the variants CYP2B6*4, *9 and *6 with BC risk.^6^

To assess the relevance of altered T metabolism, we tested whether CYP2B6‐derived T metabolites can serve as substrates for aromatase, potentially increasing oestrogenic activity and influencing oestrogen receptor‐positive BC. We confirmed that 16α‐OH‐T is converted to E3 and, for the first time, showed that 16β‐OH‐T is metabolized to 16‐epiestriol. Although underexplored, 16‐epiestriol has similar oestrogen receptor affinity to E3^38^ but appears to contribute less to overall oestrogenic activity. In postmenopausal women, serum levels of 16‐epiestriol are ~30‐fold lower than E3 and ~14‐fold in premenopausal women.^39^ Several studies have examined possible correlations of E3 and 16‐epiestriol with BC risk, which in part indicate existing associations for both, but the available data are controversial.^40^, ^41^, ^42^ Thus, it remains unknown if these metabolites impact BC development, and whether there is a difference in their prognostic impact. However, the altered metabolite profile of T that we found for all examined CYP2B6 variants can lead to a steroid hormone misbalance in vivo. This may contribute to an elevated BC risk, which was observed for carriers of these variants.^6^ To translate these findings into a clinical setting, one approach would be to measure plasma concentrations of the CYP2B6‐generated T metabolites in individuals genotyped for the respective CYP2B6 variants. Such analyses could validate these variant‐specific effects under in vivo conditions.

It is important to note that in general CYP2B6 seems to be lower expressed in BC cells compared to CYP enzymes being primarily extrahepatically active in steroid metabolism, such as CYP1B1.^43^ Therefore, the overall impact of CYP2B6 in steroid hormone metabolism in BC tissue might be less pronounced. However, the mentioned variants mostly leading to low activity of CYP2B6 have been shown to be associated with BC risk,^6^ probably due to alterations in steroid hormone metabolism. In the future, it may be studied in which context in BC treatment pharmacogenetic profiling of CYP2B6 could be meaningful. For BC risk screening in primary prevention the influence of CYP2B6 polymorphisms on steroid hormone metabolism may be only one small factor. However, in secondary prevention of relapse, such as during endocrine therapy, CYP2B6 testing might be considered to be studied in prospective clinical trials for its usefulness.

In summary, the four most prevalent CYP2B6 variants, being associated with adverse BC outcomes,^6^, ^7^ alter T metabolism when overexpressed in T47D cells. Moreover, our supersomes analyses highlight distinct T and E2 metabolite profiles for CYP2B6 *vs*. CYP1B1, potentially explaining their opposing associations with BC.^4^, ^23^ We confirm that 16α‐OH‐T is converted to E3 and newly demonstrate 16β‐OH‐T conversion to 16‐epiestriol by aromatase. As aromatase is highly active in breast tissue and both oestrogen metabolites are indicated to be linked to BC risk, a variant‐induced shift of the 16α/16β‐OH‐T metabolite ratio, could represent a potential risk factor. Our findings offer new insights into the role of CYP2B6 variants in T metabolism and their potential impact on hormone‐driven BC development.

## 5 STRENGTHS AND LIMITATIONS

Stable in vitro expression systems like T47D cells are invaluable for functional studies, and to evaluate genotype‐dependent impacts on the enzymatic activity. They enable the reduction of disturbing factors, high comparability, reproducibility and normalization, suggesting that our findings in T47D cells would be robust in various hormone‐sensitive BC cell lines of different origins. However, these systems are limited in addressing factors such as age or gender, and do not replicate genotype‐dependent expression differences found in vivo. Given that the CYP2B6*6 genotype is associated with reduced protein expression, the elevated 16α‐hydroxylation that we observed in vitro may be attenuated in vivo due to lower enzyme levels. Nonetheless, our data newly show that overexpression of CYP2B6*4, *5, *6 and *9 altered the 16α/16β‐OH‐T ratio in a human BC model. To assess transferability of results from in vitro investigations to clinical conditions, in vivo studies are necessary.

## AUTHOR CONTRIBUTIONS

Marco Hoffmann and Julia Carolin Stingl conceived and designed the study. Marco Hoffmann, Julian Peter Müller, Sabrina Yamoune and Stefan Düsterhöft performed the experiments and acquired the data. Marco Hoffmann, Julian Peter Müller and Julia Carolin Stingl analysed and interpreted the data. Marco Hoffmann and Julia Carolin Stingl drafted the manuscript. All authors (Marco Hoffmann, Julian Peter Müller, Stefan Düsterhöft, Sabrina Yamoune, Katja Susanne Just and Julia Carolin Stingl) critically revised the manuscript for important intellectual content and approved the final version to be published. All authors agree to be accountable for all aspects of the work.

## CONFLICT OF INTEREST STATEMENT

None of the authors declared any conflict of interest with the work that has been published in this article.

## Supporting information


**Data S1.** Supporting Information.

## Data Availability

Data will be made available on request.

## References

[1] Hedrich WD , Hassan HE , Wang H . Insights into CYP2B6‐mediated drug‐drug interactions. Acta Pharm Sin B. 2016;6(5):413‐425. doi:10.1016/j.apsb.2016.07.016 27709010 PMC5045548

[2] Kumagai J , Fujimura T , Takahashi S , et al. Cytochrome P450 2B6 is a growth‐inhibitory and prognostic factor for prostate cancer. Prostate. 2007;67(10):1029‐1037. doi:10.1002/pros.20597 17455229

[3] Hellmold H , Rylander T , Magnusson M , Reihnér E , Warner M , Gustafsson JA . Characterization of cytochrome P450 enzymes in human breast tissue from reduction mammaplasties. J Clin Endocrinol Metab. 1998;83(3):886‐895. doi:10.1210/jcem.83.3.4647 9506744

[4] Hlaváč V , Brynychová V , Václavíková R , et al. The role of cytochromes P450 and aldo‐keto reductases in prognosis of breast carcinoma patients. Medicine (Baltimore). 2014;93(28):e255. doi:10.1097/MD.0000000000000255 25526449 PMC4603110

[5] Kurosaki T , Suzuki M , Enomoto Y , et al. Polymorphism of cytochrome P450 2B6 and prostate cancer risk: a significant association in a Japanese population. Int J Urol. 2009;16(4):364‐368. doi:10.1111/j.1442-2042.2009.02263.x 19425200

[6] Justenhoven C , Pentimalli D , Rabstein S , et al. CYP2B6*6 is associated with increased breast cancer risk. Int J Cancer. 2014;134(2):426‐430. doi:10.1002/ijc.28356 23824676 PMC3883876

[7] Kuo S‐H , Yang S‐Y , You S‐L , et al. Polymorphisms of ESR1, UGT1A1, HCN1, MAP 3K1 and CYP2B6 are associated with the prognosis of hormone receptor‐positive early breast cancer. Oncotarget. 2017;8(13):20925‐20938. doi:10.18632/oncotarget.14995 28178648 PMC5400556

[8] PharmGKB gene‐specific information tables for CYP2B6. https://www.pharmgkb.org/page/cyp2b6RefMaterials

[9] Zhou Y , Ingelman‐Sundberg M , Lauschke VM . Worldwide distribution of cytochrome P450 alleles: a meta‐analysis of population‐scale sequencing projects. Clin Pharmacol Ther. 2017;102(4):688‐700. doi:10.1002/cpt.690 28378927 PMC5600063

[10] Mangó K , Kiss ÁF , Fekete F , Erdős R , Monostory K . CYP2B6 allelic variants and non‐genetic factors influence CYP2B6 enzyme function. Sci Rep. 2022;12(1):2984. doi:10.1038/s41598-022-07022-9 35194103 PMC8863776

[11] Sridar C , Snider NT , Hollenberg PF . Anandamide oxidation by wild‐type and polymorphically expressed CYP2B6 and CYP2D6. Drug Metab Dispos. 2011;39(5):782‐788. doi:10.1124/dmd.110.036707 21289075 PMC3082373

[12] Zanger UM , Klein K , Saussele T , Blievernicht J , Hofmann MH , Schwab M . Polymorphic CYP2B6: molecular mechanisms and emerging clinical significance. Pharmacogenomics. 2007;8(7):743‐759. doi:10.2217/14622416.8.7.743 17638512

[13] Crane AL , Klein K , Zanger UM , Olson JR . Effect of CYP2B6*6 and CYP2C19*2 genotype on chlorpyrifos metabolism. Toxicology. 2012;293(1–3):115‐122. doi:10.1016/j.tox.2012.01.006 22281205 PMC3935332

[14] Imaoka S , Yamada T , Hiroi T , et al. Multiple forms of human P450 expressed in *Saccharomyces cerevisiae*. Systematic characterization and comparison with those of the rat. Biochem Pharmacol. 1996;51(8):1041‐1050. doi:10.1016/0006-2952(96)00052-4 8866826

[15] Lee AJ , Cai MX , Thomas PE , Conney AH , Zhu BT . Characterization of the oxidative metabolites of 17beta‐estradiol and estrone formed by 15 selectively expressed human cytochrome p450 isoforms. Endocrinology. 2003;144(8):3382‐3398. doi:10.1210/en.2003-0192 12865317

[16] Kaaks R , Rinaldi S , Key TJ , et al. Postmenopausal serum androgens, oestrogens and breast cancer risk: the European prospective investigation into cancer and nutrition. Endocr Relat Cancer. 2005;12(4):1071‐1082. doi:10.1677/erc.1.01038 16322344

[17] Clemons M , Goss P . Estrogen and the risk of breast cancer. N Engl J Med. 2001;344(4):276‐285. doi:10.1056/NEJM200101253440407 11172156

[18] Key TJ , Appleby PN , Reeves GK , et al. Sex hormones and risk of breast cancer in premenopausal women: a collaborative reanalysis of individual participant data from seven prospective studies. Lancet Oncol. 2013;14(10):1009‐1019. doi:10.1016/s1470-2045(13)70301-2 23890780 PMC4056766

[19] Uno Y , Uehara S , Yamazaki H . Polymorphisms of cytochrome P450 2B6 (CYP2B6) in cynomolgus and rhesus macaques. J Med Primatol. 2018;47:232‐237 doi:10.1111/jmp.12336 29468688

[20] Canick JA , Ryan KJ . Cytochrome P‐450 and the aromatization of 16alpha‐hydroxytestosterone and androstenedione by human placental microsomes. Mol Cell Endocrinol. 1976;6(2):105‐115. doi:10.1016/0303-7207(76)90010-1 1001810

[21] Li F , Zhu W , Gonzalez FJ . Potential role of CYP1B1 in the development and treatment of metabolic diseases. Pharmacol Ther. 2017;178:18‐30. doi:10.1016/j.pharmthera.2017.03.007 28322972 PMC5600638

[22] Hayes CL , Spink DC , Spink BC , Cao JQ , Walker NJ , Sutter TR . 17 beta‐estradiol hydroxylation catalyzed by human cytochrome P450 1B1. Proc Natl Acad Sci U S a. 1996;93(18):9776‐9781. doi:10.1073/pnas.93.18.9776 8790407 PMC38505

[23] Goth‐Goldstein, R. , Erdmann, C. , & Russell, M. 2001 CYP1B1 Expression, a Potential Risk Factor for Breast Cancer. Lawrence Berkeley National Laboratory.

[24] Hollis PR , Mobley RJ , Bhuju J , Abell AN , Sutter CH , Sutter TR . CYP1B1 augments the mesenchymal, claudin‐low, and chemoresistant phenotypes of triple‐negative breast cancer cells. Int J Mol Sci. 2022;23(17):9670 doi:10.3390/ijms23179670 36077068 PMC9456208

[25] Liehr JG , Fang WF , Sirbasku DA , Ari‐Ulubelen A . Carcinogenicity of catechol estrogens in Syrian hamsters. J Steroid Biochem. 1986;24(1):353‐356. doi:10.1016/0022-4731(86)90080-4 3009986

[26] Liehr JG , Ricci MJ . 4‐Hydroxylation of estrogens as marker of human mammary tumors. Proc Natl Acad Sci U S A. 1996;93(8):3294‐3296. doi:10.1073/pnas.93.8.3294 8622931 PMC39600

[27] Fernandez SV , Russo IH , Russo J . Estradiol and its metabolites 4‐hydroxyestradiol and 2‐hydroxyestradiol induce mutations in human breast epithelial cells. Int J Cancer. 2006;118(8):1862‐1868. doi:10.1002/ijc.21590 16287077

[28] Düsterhöft S , Kahveci‐Türköz S , Wozniak J , et al. The iRhom homology domain is indispensable for ADAM17‐mediated TNFα and EGF receptor ligand release. Cell Mol Life Sci. 2021;78(11):5015‐5040. doi:10.1007/s00018-021-03845-3 33950315 PMC8233286

[29] Yamoune S , Müller JP , Langmia IM , Scholl C , Stingl JC . Uncoupling of cytochrome P450 2B6 and stimulation of reactive oxygen species production in pharmacogenomic alleles affected by interethnic variability. Biochim Biophys Acta Gen Subj. 2024;1868(5):130595. doi:10.1016/j.bbagen.2024.130595 38467309

[30] Degasperi A , Birtwistle MR , Volinsky N , Rauch J , Kolch W , Kholodenko BN . Evaluating strategies to normalise biological replicates of Western blot data. PLoS ONE. 2014;9(1):e87293. doi:10.1371/journal.pone.0087293 24475266 PMC3903630

[31] Alexander SPH , Cidlowski JA , Kelly E , et al. The concise guide to PHARMACOLOGY 2023/24: nuclear hormone receptors. Br J Pharmacol. 2023;180(Suppl 2):S223‐S240. doi:10.1111/bph.16179 38123152 PMC13480401

[32] Alexander SPH , Fabbro D , Kelly E , et al. The concise guide to PHARMACOLOGY 2023/24: enzymes. Br J Pharmacol. 2023;180(Suppl 2):S289‐S373. doi:10.1111/bph.16181 38123154

[33] Alexander SPH , Christopoulos A , Davenport AP , et al. The concise guide to PHARMACOLOGY 2023/24: G protein‐coupled receptors. Br J Pharmacol. 2023;180(Suppl 2):S23‐S144. doi:10.1111/bph.16177 38123151 PMC13324819

[34] Ketteler R , Glaser S , Sandra O , Martens UM , Klingmüller U . Enhanced transgene expression in primitive hematopoietic progenitor cells and embryonic stem cells efficiently transduced by optimized retroviral hybrid vectors. Gene Ther. 2002;9(8):477‐487. doi:10.1038/sj.gt.3301653 11948372

[35] Schneider J , Huh MM , Bradlow HL , Fishman J . Antiestrogen action of 2‐hydroxyestrone on MCF‐7 human breast cancer cells. J Biol Chem. 1984;259(8):4840‐4845. 6325410

[36] Lin H‐L , Zhang H , Kenaan C , Hollenberg PF . Roles of residues F206 and V367 in human CYP2B6: effects of mutations on androgen hydroxylation, mechanism‐based inactivation, and reversible inhibition. Drug Metab Dispos. 2016;44(11):1771‐1779. doi:10.1124/dmd.116.071662 27538916 PMC5074475

[37] Uno Y , Matsuno K , Nakamura C , Utoh M , Yamazaki H . Identification and characterization of CYP2B6 cDNA in cynomolgus macaques (*Macaca fascicularis*). J Vet Med Sci. 2009;71(12):1653‐1656. doi:10.1292/jvms.001653 20046035

[38] Raynaud JP , Ojasoo T , Bouton MM , Philibert D . Receptor Binding as a Tool in the Development of New Bioactive Steroids Drug Design. Elsevier; 1979:169‐214.

[39] Coburn SB , Stanczyk FZ , Falk RT , et al. Comparability of serum, plasma, and urinary estrogen and estrogen metabolite measurements by sex and menopausal status. Cancer Causes Control. 2019;30(1):75‐86. doi:10.1007/s10552-018-1105-1 30506492 PMC6447065

[40] Eliassen AH , Spiegelman D , Xu X , et al. Urinary estrogens and estrogen metabolites and subsequent risk of breast cancer among premenopausal women. Cancer Res. 2012;72(3):696‐706. doi:10.1158/0008-5472.CAN-11-2507 22144471 PMC3271178

[41] Dallal CM , Tice JA , Buist DSM , et al. Estrogen metabolism and breast cancer risk among postmenopausal women: a case‐cohort study within B~FIT. Carcinogenesis. 2014;35(2):346‐355. doi:10.1093/carcin/bgt367 24213602 PMC3908751

[42] Falk RT , Brinton LA , Dorgan JF , et al. Relationship of serum estrogens and estrogen metabolites to postmenopausal breast cancer risk: a nested case‐control study. Breast Cancer Res. 2013;15(2):R34. doi:10.1186/bcr3416 23607871 PMC4053199

[43] Sneha S , Baker SC , Green A , et al. Intratumoural cytochrome P450 expression in breast cancer: impact on standard of care treatment and new efforts to develop tumour‐selective therapies. Biomedicine. 2021;9(3):1‐22 doi:10.3390/biomedicines9030290 PMC7998590 33809117

