# Supplementary material for: Pharmacogenetic CYP2B6 variants affect steroid hormone metabolism in human breast cancer cells
Source: Br J Clin Pharmacol. 2026 Feb 4;92(7):2075–84. doi: 10.1002/bcp.70473 (PMC13304254; doi:10.1002/bcp.70473)
Supplement: Supplementary file 1 — Data S1. Supporting Information. [file BCP-92-2075-s001.docx]

**Supplementary file 1**

**Material & methods (in detail descriptions)**

**Materials**

T47D cells (HTB-133) were purchased from ATCC (Manassas, Virginia, USA) and authenticated by STRS analysis. Ultra Gradient HPLC Grade acetonitrile (9017) and methanol (8402) were purchased from J.T. Baker (Gliwice, Poland). Ammonia solution 25% (5.33003.0050) and LC-MS grade water (1.15333.2500) were from Merck (Darmstadt, Germany) and formic acid (84865.180) was from VWR International (Leuven, Belgium). Dimethyl sulfoxide (DMSO) (A994.2), 2-(4-(2-hydroxyethyl)-1-piperazinyl)-ethansulfonic acid (HEPES) (9105.4), tris-base (4855.2) and milk powder (T145.3) were purchased from Carl Roth (Karlsruhe, Germany). Testosterone (86500), estradiol (E2758), L-Ascorbic acid (A4544), 4-OH-E2 (H4637), estriol (E1253), Phenylmethylsulfonylfluorid (PMSF) (10837091001), Dulbeccos’s Phosphate Buffered Saline (PBS) (D8537), Roswell Park Memorial Institute (RPMI) medium (R7388), Dulbeccos modified Eagle-medium (DMEM) (D6429), trypsin (T3924) and penicillin-streptomycin solution (P/S) (P4333) were from Sigma Aldrich (St. Louis, Missouri). 2-OH-E2 (13019-1) was purchased from Cayman Chemicals (Ann Arbor, Michigan, USA). Fetal bovine serum (FBS) (P40-37500) was from PAN Biotech (Aidenbach, Germany). Nicotinamide adenine dinucleotide phosphate (NADPH) (16156.500), 16-epiestriol (Cay33455-10) and puromycin (Cay13884-100) were from Biomol (Hamburg, Germany). Metabolites 16α- (AAA06301) and 16β-OH-T (3D-SAA52890) were purchased from Biosynth (Staad, Switzerland). 4x Laemmli buffer (1610747), Precision Plus Protein™ Dual Color Standards (Marker) (1610394), 4–15% Mini-PROTEAN® TGX™ Precast Protein Gels (4561083), Mini-Protean Tetra Cell (1658004), Mini Trans-Blot Module (1703935), 10x Tris/Glycine/SDS (electrophoresis buffer) (1610772), 10x Tris/Glycin (transfer buffer) (1610771), 10x Tris Buffered Saline (TBS) (1706435), Immun-Blot polyvinylidene fluoride (PVDF) Membrane (1620177) 10% Tween 20 (1610781) and Clarity Western ECL (enhanced chemiluminescence) Substrate (1705062) were from BioRad (Hercules, California, USA). Agarose (840001) was purchased from Biozym (Hessisch Oldendorf, Germany). DpnI (R0176S) Pacl (R0547S) and EcoRI (R3101S) were from New England Biolabs (NEB) (Ipswich, Massachussets, USA).

**Testosterone and estradiol metabolism by isolated CYP2B6 and CYP1B1**

For the examination of T and E2 metabolite profiles, CYP2B6 (Order no.: 456210) or CYP1B1 (Order no.: 456220) supersomes (Discovery Life Sciences, Kassel, Germany) were used. Specifically, For T or E2 experiments, 140 pmol or 70 pmol of supersomes were mixed with 100 µM of T or 20 µM of E2 and 2 mM NADPH in 0.1 M Tris-HCl buffer at pH 7.4 and 0.05 M HEPES in a final volume of 100 µl. To stabilize the hydroxylated E2 metabolites, a final concentration of 5 mM ascorbic acid was added to the E2 approaches. Mixtures were incubated at 37 °C at 300 rpm for 1 hour (h) and the reaction was stopped by adding 400 µl of 4 °C cold methanol. After mixing thoroughly, the samples were centrifuged at 17.000 x *g* at 4 °C for 20 minutes (min) and 100 µl of the supernatants were measured via HPLC-MS/MS.

**Choice of cell line for CYP2B6-vector transduction**

For vector transduction, we tested the two cell lines MCF-7 and T47D, since both are well researched and commonly used for CYP enzyme overexpression studies, and exhibit the same BC subtype. MCF-7 cells were poorly transducable and mostly died, while T47D cells showed a strong and persistent overexpression of the target gene. We therefore considered T47D cells to be the suitable model for our investigations.

**pMOWS vector cloning for T47D cell transduction**

For stable *CYP2B6* overexpression in T47D cells, the pMOWS vector system [1] with expression of green fluorescence protein (*GFP*) as reporter gene, was used. To increase protein expression, a cytomegalovirus-promotor was integrated upstream of the *GFP* gene and for antibiotic selection a puromycin resistance gene was introduced. The cDNA sequences of the variants CYP2B6*1, *4, *6 and *9 were cloned from a pcDNA3.1 vector [2] using CloneAmp Hifi PCR Mix (Order no.: 639298, Takara, Shiga, Japan) according to the manufacturer`s instructions. The forward primer used to amplify the inserts with pMOWS vector overhangs was 5’- AGGCGCCACCGCGGTGGTTAATTAACCATGGAACTCAGC-3’ and the reverse primer sequence was 5’-TGTAATCCAGAGGTTGATTGAATTCTCAGCGGGGCAGGA-3’. *CYP2B6*5* was generated using the mutagenic reverse primer 5’- GGTT GATTGAATTCTCAGCGGGGCAGGAAGCAGATCTGGTATGTT-3’ in a PCR with CYP2B6 wild type cDNA. 5 µl of the PCR products were subjected to electrophoresis in a 1% agarose gel stained with SYBR Safe Gel Stain (Order no.: S33102, ThermoFisher Scientific, Waltham, Massachusetts). The PCR products were digested with 20 units of DpnI and purified using a DNA-CleanUp kit (Order no.: T1030S, NEB). 10 µg of the target vector pMOWS was linearized excising the integrated *GFP*-sequence by using 5 units of PacI and EcoRI. The linearized vector was then subjected to a 1% agarose gel electrophoresis and the respective gel band (~6000 bp) was extracted by a gel extraction kit (Order no.: T1020S, NEB). Afterwards, Gibson Assembly was performed with 50 ng of the vector and 100 ng of the respective insert for 20 min at 50 °C using Hifi DNA Assembly Master Mix (Order no.: E2621L, NEB). The pMOWS vectors containing the different CYP2B6 genotypes were then transformed in *E. coli* (Order no.: C3040I, NEB), single colonies were picked, and DNA was isolated using a Midi-prep kit (Order no.: 740410.50, Macherey-Nagel, Düren, Germany). Vector sequencing was performed for all constructs by Eurofins Genomics using Sanger sequencing, to check for successful cloning.

**Generation of stable T47D cells overexpressing GFP or CYP2B6 variants**

First the retroviruses containing the respective target gene sequences (*GFP*, *CYP2B6*1*, **4*, **5*, **6*, *or *9*) were produced. Therefore, the Phoenix-AMPHO retrovirus producer cell line for generation of helper-free ecotropic and amphotropic retroviruses was used. In the first step, 10 x 10^6^ Phoenix cells were seeded in DMEM medium containing 10% FBS and 1% P/S onto a 100 mm culture dish and immediately transfected with the pMOWS vector containing the respective target gene sequence using Lipofectamine 3000 (Order no.: L3000015, ThermoFisher Scientific) according to the manufacturer’s protocol. After 24 h, the medium was exchanged for 8 ml of fresh DMEM and incubated for another 24 h. The medium containing the produced retroviruses was sterile filtered (pore size: 0.45 µm). Then, 8 ml of the filtrate were mixed with 5 ml of fresh RPMI medium (10% FBS, 1% P/S) and added to currently seeded 3 x 10^6^ T47D cells in a new 100 mm culture dish for transduction. 13 µl of polybrene were also added to the dish, before carefully mixing. Based on previous kill-curves of T47D and puromycin ranging from 0.05 to 10 µg/ml, 0.5 µg/ml were found to be suitable for the selection of isogenic cells. Therefore, after 48 h, the selection was started by changing the medium to RPMI with 0.5 µg/ml of puromycin. Selection medium was exchanged every 2-3 days for 2 weeks. All cells were maintained at 37 °C in a 5% CO_2_ environment.

**Quantitative real-time polymerase chain reaction (qPCR) measurement of *CYP2B6* overexpression in T47D cells**

For verification of the mRNA overexpression of the different *CYP2B6* variants in transduced T47D cells, qPCR was carried out. 1 x 10^6^ cells were seeded into 6-well plates 24 h before harvesting by using 1 ml of *TRIzol^TM^* reagent (Order no.: 15596-018, ThermoFisher Scientific) and RNA was isolated according to the manufacturer´s protocol. 1 µg of RNA per sample was used for cDNA synthesis using *High-Capacity cDNA Reverse Transcription Kit* (Order no.: 4368814, ThermoFisher Scientific) according to the manufacturer´s protocol. qPCR was carried out using *PowerUP SYBR Green* (Order no.: A25776, ThermoFisher Scientific) with *QuantiTect Primer Assays* (QIAGEN, Venlo, Netherlands) as described in the manufacturer´s protocol. Primer assays for the genes of phosphoglycerate kinase 1 (*PGK1)* (Order no.: QT00013776) and *CYP2B6* (Order no.: QT00000910) were used. *PGK1* gene expression measurement was used for normalization of the *CYP2B6* cycle threshold (Ct) values for quantification.

**Western blot analysis of CYP2B6 overexpression in T47D cells**

CYP2B6 protein expression in transduced T47D cells was examined by western blot analysis. Therefore, 5 x 10^6^ cells were seeded onto 60 mm dishes 24 h before cell lysis. The cells were washed with ice-cold PBS and then incubated for 30 min on a rotating plate at 4 °C in 600 µl lysis buffer (50 mM Tris, 137 mM NaCl, 2 mM EDTA, 1% Triton X-100, pH 7.5) with added Phosphatase inhibitor cocktails II, III and Protease inhibitor cocktail (Order no.: HY-K0022, HY-K0023, HY-K0011, MedChemExpress, Monmouth Junction, New Jersey) as recommended in manufacturer´s protocols and 1 mM PMSF to prevent protein degradation. Lysates were then centrifuged at 10.000 x *g*, 4 °C for 15 min, the supernatant was mixed with Laemmli buffer as loading dye and incubated at 95 °C for 5 min for protein denaturation. Marker, samples and 0.5 pmol of CYP2B6 supersomes as positive control were loaded onto the electrophoresis gel and the run was started. Afterwards, proteins were transferred onto a PVDF membrane for 1 h. Membranes were washed with 1x TBS containing 0.1% Tween20 (TBS-T) and blocked using 5% milk in TBS-T for 1 h. Primary rabbit anti-human polyclonal CYP2B6 antibody (Order no.: 70R-32408, Biosynth, Staad, Switzerland) was solved in 1% milk in TBS-T (final concentration 1.4 µg/ml) and incubated for about 20 h at 4 °C. Secondary goat anti-rabbit IgG horseradish-peroxidase (HRP) antibody (Order no.: 31460, ThermoFisher Scientific) was solved in 0.1% milk in TBS-T (final concentration 80 ng/ml) and incubated for 1 h at room temperature. For imaging, the membranes were incubated for 3 min in ECL substrate (BioRad). Detection of glycerinaldehyde 3-phosphat dehydrogenase (GAPDH) took place by incubation in 1% milk-TBS-T diluted primary mouse anti-human monoclonal antibody (Order no.: 649202, Biolegend, San Diego, CA) (final concentration 0.5 µg/ml) for about 20 h at 4 °C. Afterwards, secondary goat anti-mouse IgG HRP antibody (Order no.: 31569, ThermoFisher Scientific) in 0.1% milk (final concentration 0.75 µg/ml) was incubated for 1 h at room temperature. Each repeated western blot experiment reflects a different passage of the cells. The mean relative expression ratio of the different CYP2B6 variants was calculated by division of each band intensity by the sum of all band’s intensity on each blot, described by Degasperi et al. [3]. GAPDH detection served as loading control and band intensity was used for CYP2B6 expression normalization. Calculated CYP2B6 expression ratio was used for normalization of T metabolism experiments in T47D cells.

**Testosterone metabolism in T47D cells overexpressing CYP2B6 variants**

Between experiments T47D cells were cultivated in RPMI medium containing 0.5 µg/ml puromycin for maintaining selective pressure on the transduced cells. 2-3 days before cell seeding, medium was changed to RPMI without puromycin to reduce cell stress during the experiment. Medium was changed every 2-3 days and cells were subcultured at 80-90% confluency using trypsin reagent. For T metabolism experiments, the same cell passages as in the western blot experiments were used (passages 1 – 3). The CYP2B6-variants overexpressing T47D cells were seeded into 6-well plates with 2.4 x 10^6^ cells per well. After 24 h, the medium was exchanged to RPMI without FBS containing 100 μM of T (Stock: 10 mM; final DMSO concentration: 1%), to start the reaction. After 1, 4, 8 and 16 h, 50 µl of the supernatants were collected and thoroughly mixed with 200 µl of 4 °C cold methanol. All samples were centrifuged at 17.000 x *g* and 4 °C for 20 min and 100 µl of the supernatants measured via HPLC-MS/MS. To enable quantitative comparisons between the different CYP2B6 variants expressing cells, the formed metabolite levels were normalized to the total protein content measured using a bicinchoninic acid (BCA)-assay. The enzymatic activity of the different variants was assessed by calculating the relative metabolite formation rate through dividing the rate of T metabolism (pmol/min) by the CYP2B6 expression ratio determined by the western blot experiments.

**Bicinchoninic Acid (BCA) assay of T47D samples**

To normalize the T metabolite levels derived from the T47D cell experiments to total protein content the bicinchoninic acid (BCA) assay (Order no.: 23225, ThermoFisher Scientific) was used. Therefore, after supernatant collection the cells were washed two times with ice cold PBS and were then incubated for 30 min on a rotating plate at 4 °C in 250 µl lysis buffer (50 mM Tris, 137 mM NaCl, 2 mM EDTA, 1% Triton X-100, pH 7.5). The lysates were centrifuged at 10.000 x *g*, 4 °C for 15 min and BCA-assay was performed according to the manufacturer’s protocol using the supernatant of the samples.

**Conversion of testosterone metabolites to estriol or 16-epiestriol by aromatase**

For investigation of 16α- and 16β-OH-T conversion to E3 or 16-epiestriol by aromatase, 70 pmol of isolated aromatase supersomes (Order no.: 456260, Discovery Life Sciences) were mixed with 2 mM of NADPH and 20 µM of 16α- or 16β-OH-T in 0.1 M Tris-HCL buffer with 0.05 M HEPES at pH 7.4 with a final volume of 100 µl. Mixtures were incubated at 37 °C with 300 rpm for 1 h and the reaction was stopped by adding 400 µl of 4 °C cold methanol. After mixing thoroughly, the samples were centrifuged at 17.000 x *g* at 4 °C for 20 min and 100 µl of the supernatants were measured via HPLC-MS/MS.

**High performance liquid chromatography – tandem mass spectrometry (HPLC-MS/MS) measurements of steroid hormone metabolites**

HPLC-MS/MS analysis was carried out using an Agilent 1290 Infinity II UHPLC coupled to a SCIEX QTRAP6500+ triple quadrupole mass spectrometer.

2-OH-E2 and 4-OH-E2 were measured on an Agilent Poroshell 120 Phenyl Hexyl column (2.7 µm, 2.1 x 50 mm, Order no.: 699775-912, Agilent Technologies, Santa Clara, USA) with A: 0.1% (v/v) formic acid in water and B: 0.1% (v/v) formic acid in acetonitrile. The injection volume was 5 µl and the LC flow rate was 0.6 ml/min with a total run length of 7 min. The LC gradient was as follows: 0 min 10% B, 0.1 min 10% B, 4 min 30% B, 4.3 min 95% B, 5.3 min 95% B, 5.5 min 10% B and 7 min 10% B. 2-OH-E2 and 4-OH-E2 were measured with negative ion multiple reaction monitoring (MRM) mode with the mass transitions 287 🡪 161 *m/z* (quantification, collision energy (CE): -56 V), 287 🡪 199 *m/z* (qualification, CE: -54 V) and 287 🡪 147 *m/z* (qualification, CE: -56 V).

16α-OH-T and 16β-OH-T were measured on an Agilent Poroshell 120 EC-C18 column (1.9 µm, 2.1 x 50 mm, Order no.: 699675-902, Agilent Technologies) with A: 0.1% (v/v) formic acid in water and B: methanol. The injection volume was 5 µl. The LC flow rate was 0.6 ml/min with a total run length of 7 min. The gradient was as follows: 5% B at 0 min, 95% B at 4.5 min, 95% B at 5 min, 5% B at 5.2 min, 5% B at 7 min. 16α-OH-T and 16β-OH-T were measured in positive ion MRM mode with the mass transitions 305 🡪 97 *m/z* (quantification) and 305 🡪 109 *m/z* (qualification) with a collision energy of 29 V.

E3 and 16-epiestriol were measured in negative ion mode using a Kinetex EVO C18 column (2.6 µm, 2.1 x 50 mm, Order no.: 00B-4725-AN, Phenomenex, Torrance, USA) with A: 10 mM ammonium acetate buffer at pH 9 in water and B: methanol. MRM mass transitions to quantify E3 and 16-epiestriol were 287 🡪 171 *m/z* (quantification, CE: -48 V), 287 🡪 145 *m/z* (qualification, CE: -15 V) and 287 🡪 143 *m/z* (qualification, CE: -17 V). The LC flow rate was 0.4 ml/min with a total run length of 4 min. The injection volume was 10 µl. The gradient was as follows: 5% B at 0 min, 90% B at 1.5 min, 90% B at 2.5 min, 5% B at 2.7 min, 5% B at 4 min.

For quantification of the specific analytes, the analyte peaks were integrated and quantified using external calibration curves. Calibration curves of the respective analytes were constructed in sample matrix with a minimum of 5 non-zero standards. The ranges of the calibration curves were applied as follows: From 0.05 to 500 ng/ml for 16α- and 16β-OH-T and from 0.1 to 100 ng/ml for 2- and 4-OH-E2, as well as for E3 and 16-epiestriol.

Literatur

1. Düsterhöft S, Kahveci-Türköz S, Wozniak J, Seifert A, Kasparek P, Ohm H, Liu S, Kopkanova J, Lokau J, Garbers C, Preisinger C, Sedlacek R, Freeman M, Ludwig A (2021) The iRhom homology domain is indispensable for ADAM17-mediated TNFα and EGF receptor ligand release. Cell Mol Life Sci 78(11):5015–5040. doi:10.1007/s00018-021-03845-3

2. Yamoune S, Müller JP, Langmia IM, Scholl C, Stingl JC (2024) Uncoupling of Cytochrome P450 2B6 and stimulation of reactive oxygen species production in pharmacogenomic alleles affected by interethnic variability. Biochim Biophys Acta Gen Subj 1868(5):130595. doi:10.1016/j.bbagen.2024.130595

3. Degasperi A, Birtwistle MR, Volinsky N, Rauch J, Kolch W, Kholodenko BN (2014) Evaluating strategies to normalise biological replicates of Western blot data. PLoS One 9(1):e87293. doi:10.1371/journal.pone.0087293
